# Making the 2007-2010 Action Plan work for women in family medicine in the Asia Pacific

**DOI:** 10.1186/1447-056X-9-1

**Published:** 2010-01-07

**Authors:** Jan Coles, Amanda Barnard, Amanda Howe, Jo Wainer, Zorayda Leopando, Sarah Strasser

**Affiliations:** 1 Department of General Practice, Monash University, Australia; 2 School of General Practice, Rural and Indigenous Health, Head, Rural Clinical School, Australian National University, Australia; 3 School of Medicine Health Policy and Practice, University of East Anglia, UK; 4 Gender and Medicine Research Unit, Faculty of Medicine, Nursing and Health Sciences, Monash University, Australia; 5 Department of Planning and Development/Family and Community Medicine, University of the Philippines, Manila, Philippines; 6 Faculty Affairs Unit, Northern Ontario School of Medicine, Canada

## Abstract

The Wonca Working Party for Women and Family Medicine (WWPWFM) was organized in 2001 with the following objectives: to identify the key issues for women doctors; to review Wonca policies and procedures for equity and transparency; to provide opportunities to network at meetings and through the group's listserve and website; and to promote women doctors' participation in Wonca initiatives.

In October 2008, at the Asia Pacific Regional conference, the Wonca Working Party on Women in Family Medicine (WWPWFM) held a preconference day and conference workshops, building on the success and commitment to initiatives which enhance women's participation in Wonca developed in Ontario, Canada (2006) and at the Singapore World Congress (2007). At this meeting fifty women workshopped issues for women in Family Medicine in the Asia Pacific. Using the Action Plan formulated in Singapore (2007) the participants identified key regional issues and worked towards a solution.

Key issues identified were professional issues, training in family medicine and women's health. Solutions were to extend the understanding of women's contributions to family medicine, improved career pathways for women in family medicine and improving women's participation in practices, family medicine organizations and academic meetings.

## Introduction

Gender equity is a pressing need for family medicine [1], and in the communities of the Asia Pacific. Women earn less, are more likely to live in poverty and income inequality is rising [2]. Women are less likely to be managers and legislators in most countries within the region, with the exception of the Philippines [2]. Women are an integral part of family medicine, in the professional workforce and as patients. Women doctors are increasingly represented in the family medicine workforce around the world. In some countries of the Asia Pacific Region, women are the major providers of family medical care (Philippines), in many countries the numbers of women are increasing (Australia), while in some parts of Asia there are still few women family doctors [3,4].

The health of our communities depends, in part, on the ability of the community to access appropriate medical care and the ability of the healthcare workforce to deliver the best possible care. Women still face barriers to achieving their full potential. Women doctors face barriers in training, in practice, in medical organisations and in academic medicine [3,5,6]. The lack of gender equity in medical leadership, including the historical lack of women in senior World Association of National Colleges and Academies for General Practitioners/Family Physicians (Wonca) positions, is a challenge and a concern for family medicine [1].

Best possible care in family medicine requires gender competent professionals who understand the cultural, social and political determinants of health and can respond effectively to them. Medical knowledge and research evidence must include male and female participants [7] and gendered professional perspectives to better support gender-competent care. For example, the same serious illness, like myocardial ischemia, may present differently in male and female patients and doctors need to be trained and able to recognise gender differences in presentation. Being a victim of intimate partner violence is much commoner for women than men, and has significant impacts on health, prevention, recognition and early intervention can improve health outcomes. Women, as professionals and patients, can assist the development of gender competent knowledge and healthcare.

The 1998 XVth World Congress of the Wonca in Dublin saw the foundation of the Women in Family Medicine Special Interest Group. The group audited the structures of Wonca and the conference and challenged the "invisibility" of women in leadership and plenary positions. This, and their experiences of being made invisible in their own profession, generated the momentum for change. The result was the establishment of the Wonca Working Party on Women in Family Medicine (WWPWFM) at the 16^th ^International Conference of Wonca in Durban, in 2001 [8]. The WWPWFM aims to develop policy which would enhance the role of women family doctors in Wonca and within health systems [3] through representation, training, support, research and leadership for women in family medicine.

The Wonca 2008 Asia Pacific Regional conference was hosted in Melbourne, Australia from 2-5^th ^of October and held with the Royal Australian College of General Practitioners (RACGP) Annual Scientific Meeting. The WWPWFM held a preconference day and conference workshops, building on the success and commitment to initiatives which enhance women's participation in Wonca developed in Ontario, Canada (2006) and at the Singapore World Congress (2007). This paper reports on the preconference workshop.

## Taking Action at the Asia Pacific Regional Conference in Melbourne

Local representatives in WWPWFM organised a full day pre conference workshop to continue the work of including women. The workshop was dedicated to promoting Wonca's Ten Steps to Gender Equity and Health [9] and engaging with local issues of concern for Women in Family Medicine. Fifty women doctors registered and attended the workshop, most were from Australia and New Zealand, with representatives from the Philippines, Vietnam, Thailand, Indonesia, Pakistan, India, Canada, United States of America and the United Kingdom.

Consistent with the principles of participatory action research and a democratic respect for the knowledge of women doctors, the workshop focused on the experiences of all participants. Doctors from the region identified local gender and health equity issues, and areas of need, as well as strategies to address them. These strategies are locally and internationally relevant.

## Asia Pacific Issues for Women Doctors

### Themes

Three key themes were identified by participants. They were professional issues, training and issues for women as patients.

Professional issues which emerged were

• Challenges in achieving of gender equity in the professional consultation: time, complexity, financial remuneration;

• Negative attitudes to women family practitioners focused on their gendered workload; "longer consultations", "tears and smears", "touchy feely stuff", patient-centred approach and family priorities;

• Devaluing of part time work as "not a real doctor";

• Challenge of recognising women practitioners' professional work outside the consultation e.g. teaching;

• Inequality of opportunity e.g. funding for conferences, presenting at conferences;

• Challenges attracting women practitioners e.g. to rural practice, to leadership roles;

• Lack of female and cross-cultural role models;

• Lack of clear career paths, career flexibility and support;

• Maintaining a work/life balance.

Training issues which emerged were

• Rigid training models e.g. compulsory professional training placements often involving geographic relocation, lacked of flexibility, were full time placements with limited or no access to part-time placements, trainees reported a lack of maternity leave and a lack of clear career paths and choices;

• Need to develop negotiating skills and confidence;

• Lack of female supervisors and mentors;

• Importance and facilitation of women in training when successful female supervisors and mentors were available;

• Difficulties in maintaining work/life balance, particularly in relation to professional roles with family work and childcare.

Issues for women

• Attitudes to women;

• Violence against women;

• Gender inequity in "family work".

## Towards a solution

Participants focused on solutions to professional issues. These solutions included: extending understanding of women's contributions to family medicine, valuing women's abilities, providing clear career paths and improving women's participation in practices and organisations.

### Extending understanding of women's contributions to family medicine

Changing attitudes to women's work was a key process needed to create a greater understanding of women's contributions to medicine. Many attitudes to women and their work are culturally embedded but not clearly articulated as a gender bias within medicine. Medicine has tended to reinforce the traditional views of women [10,11]. As family medical practitioners, we must first explore the assumptions underlying our profession's attitudes to gendered social and professional roles if we are to extend our understandings [12,13].

Sexism and discrimination based on the sexual stereotyping of female behavior and work is destructive and its effects were explored by workshop participants.

The women at the workshop reported their colleagues described their medical work as "second rate" because they took longer with patients and because of the type of consultations patients requested of them. They talked of complex consultations where they practiced preventive care and addressed psychosocial as well as medical issues as very important and they valued their work highly. Some were visibly upset when they described the discriminatory language they had heard from others. "Tears and smears" was the example they used to describe the systematic and professional discrimination against their skills despite complexity and difficulty of the work they performed. The medical literature supports women's professional consultation styles as improving patient outcomes [5,14-16]. As women doctors, the participants felt, it is important to have confidence in the quality of their work and its value to patients. This will help colleagues understand women's contributions and redefine "quality" in terms of patient outcomes. Participants urged their colleagues to take action. Participant's urged their colleagues to challenge others when they dismiss women doctors' professional work by responding "Yes, I do many complex consultations" and ask if they do and if they do not then why not? Valuing women and their work, will model and teach others (both male and female) to do the same and challenge old and outdated cultural attitudes.

### Improving career paths for women in family medicine

Participants felt that transparency in training was important for women doctors: knowing up front what was required and the flexibility within those requirements; in some countries part-time training and/or re-entry was not an option and participants felt that this should be reconsidered and supported. Models from countries with flexible and part-time training could be explored and adapted where necessary.

The near invisibility of women doctors as leaders, role models and mentors was explored. Participants felt women doctors in academic, professional and organizational roles were important in supporting the careers of others and assisting them to identify career paths and choices and to better manage academic and clinical work, medical politics and policy, research, teaching and family time. Institutional and medical organisational support for women as leaders and role models was identified as a key part of this modeling process. Participants suggested training in negotiation, assertiveness and leadership during their training as medical students, and in family medicine specialization, would be beneficial.

### Improving women's participation in practices and organisations

Attitudes were identified as a major barrier to women participating actively in medical organisations. The culture of medicine and the expectation that doctors are male remained a significant barrier to women's active participation. Work and organizational practices made it difficult for women to contribute for example: directive leadership styles are more valued than collaborative ones, gender differences in consultation styles are poorly understood, emotional work is barely recognised, the lack of flexibility, part-time training and maternity leave, and timing of committee and management meetings are not family friendly.

## Outcomes

Participants elected to carry two projects forward to better support women doctors of the Asia Pacific in their professional roles. The first is to contact and survey the needs of trainees and academy/college policy across the region to allow the WWPWFM to actively address trainee needs. This initiative is directly linked to the Action Plan (See below) 2007-2010 from the Triennial Wonca Meeting of the WWPWFM 24-26^th ^July 2007, Singapore to "Promote the development of young/new family physicians with the aim of overcoming the challenges and barriers to their full development". The second is an internet based leadership module based on previously run conference leadership workshops as a resource within the region for women in family medicine to develop, support and celebrate the ways in which women doctors of the Asia Pacific practice medicine. Both will be further progressed through meetings in 2009 and beyond.

## ACTION PLAN FROM TRIENNIAL WONCA MEETING WWPWFM 24-26 JULY 2007, SINGAPORE

(UPDATED version 3, 26 Aug 2007)

1. Promote implementation of Gender Equity Bylaw Amendments.

2. Ensure that Wonca 2010 (Cancun) and other Wonca regional and rural meetings have gender equitable programmes.

3. Develop a Code of Conduct for Wonca Meetings to promote equity for women and related ethical issues.

4. Support Wonca activities in ensuring FM is a speciality throughout the world.

5. Promote development of young/new family physicians with particular attention to overcoming their challenges and barriers to full development.

6. Work with the WWPRFM (Rural) to amend the Wonca guidebook to reflect a gender equity perspective.

7. Use Wonca as a forum to highlight areas where professional leaders could be influential in legislating and regulating conditions which support equity and wellbeing for female practitioners.

8. Explore, promote and translate the 10 Steps to Gender Equity and Health into the core publications and mission statement of Wonca.

9. Work within Wonca to ensure that the ICPC codes can deliver accurate information about the issues highlighted in the care of women patients.

10. Support members of the WP to showcase the ways in which their practice and research contribute to the care of women patients, by sharing these in the group and presenting related work in Wonca meetings.

11. Ensure that our website, list serve, meetings and all communications are effective and inclusive, serving the purpose for which the WWP has been set up, and progressing this vision by celebrating the work we are doing.

12. Provide mentorship to others within the group as requested, specifically the younger participants whose careers are still being developed.

13. Commit the Working Party to continue to address issues of linguistic diversity - for example, by endeavour to ensure that the core material we prepare for each regional meeting (flyers, HER statement...) is available in the language of the host organisation as well as the preferred language.

14. Create a prize for recognition of the work done by a family physician who has embodied the issues of gender and equity for women in their clinical and academic practice, and getting this awarded by Wonca as a high profile event.

15. Convene a WWPWFM meeting in 2009 to ensure that the activities of the WWPWFM continue - to review the vision, objectives, action plan, and to plan for the preconference and 'women's track' in Cancun 2010.

16. Continue to secure and distribute bursaries for travel to maximum effect, using the budget in an equitable fashion.

## Competing interests

The authors declare that they have no competing interests.

## Authors' contributions

JC workshop facilitator, analysis transcripts from workshop and principal author.

AB workshop facilitator, critique and ordering of final manuscript.

AH workshop leader, critique and amendments to manuscript.

JW workshop conceptualization, recording and preparation documents for analysis, critique analysis and history WWP.

ZL history and WWP document provision.

SS workshop facilitator.

All authors have read and approved the final manuscript.
